# On the genus *Pseudocneorhinus* (Coleoptera, Curculionidae, Entiminae), with descriptions of five new species from China

**DOI:** 10.3897/zookeys.853.33311

**Published:** 2019-06-06

**Authors:** Li Ren, Roman Borovec, Runzhi Zhang

**Affiliations:** 1 Key Laboratory of Zoological Systematics and Evolution, Institute of Zoology, Chinese Academy of Sciences, No. 1 Beichen West Road, Chaoyang District, Beijing 100101, China Institute of Zoology, Chinese Academy of Sciences Beijing China; 2 Czech University of Life Sciences Prague, Faculty of Forestry and Wood Sciences, Department of Forest Protection and Entomology, Kamýcká 1176, CZ-165 21 Praha 6-Suchdol, Czech Republic Czech University of Life Sciences Prague Prague Czech Republic; 3 University of Chinese Academy of Sciences, Beijing 100049, China University of Chinese Academy of Sciences Beijing China

**Keywords:** New taxa, parthenogenetic, taxonomy, Trachyphloeini, weevil

## Abstract

Species of the genus *Pseudocneorhinus* occurring in or near China are reviewed, with description of five new species, *Pseudocneorhinusangustus***sp. nov.**, *P.glaber***sp. nov.**, *P.hlavaci***sp. nov.**, *P.obliquehumeralis***sp. nov.**, and *P.setosicallus***sp. nov.** from the provinces of Beijing, Gansu, Shaanxi, Sichuan, and Zhejiang. They are illustrated and compared with similar species, and a key is provided to all presently known species of the genus. Lectotypes of the following species are designated: *Callirhopalussubcallosus* Voss, 1956 [current name *Pseudocneorhinussubcallosus* (Voss, 1956)] and *P.squamosus* Marshall, 1934. *Pseudocneorhinussquameus* Morimoto, 2015 is confirmed for the fauna of China.

## Introduction

The genus *Pseudocneorhinus* Roelofs, 1873 has been transferred between tribes several times. The genus was originally placed in Leptopsides Lacordaire, 1863 (Roelofs 1873), and subsequently in the tribes Eremnini Lacordaire, 1863 (Schenkling and Marshall 1931), Callirhopalini sensu Voss, 1956 (Morimoto 1962, Chao and Chen 1980), Pseudocneorhinini Kôno, 1930 (Morimoto 1989, Han et al. 2000, Morimoto et al. 2015) and Trachyphloeini Gistel, 1848 (Zherikhin and Egorov 1991, Egorov et al. 1996, Alonso-Zarazaga and Lyal 1999, Borovec 2009, 2013, Alonso-Zarazaga et al. 2017), where it is retained in this study. Alonso-Zarazaga et al. (2017) listed 14 species from China, the Russian Far East, Japan, and Korea. Species are found in a warm steppic habitat, forest litter, and also in high mountains.

Together with the species newly described herein, the genus increases to 19 species known from the same area, with *Pseudocneorhinusbifasciatus* Roelofs, 1880 having been introduced into the USA (Wheeler and Boyd 2005). The latter is comparatively well studied, with known biology, larva, and pupa (for example Allen 1959, Zepp 1978). Males are known only in nine species and the others are assumed to be parthenogenetic. Marshall (1934) and Voss (1956) studied regional collections from China, Han et al. (2000) and Han and Yoon (2000) from South Korea and Morimoto et al. (2015) from Japan.

The genus was recently redescribed by Borovec (2009). *Pseudocneorhinus* is related to genera *Rhinodontus* Faust, 1890 and *Rhinodontodes* Voss, 1967 sharing with them the ocular lobe in the lateral part of anterior pronotal margin, but it differs from both by the ocular lobe without setae, the rostrum lacking a lengthened epistome in females, the antennal scape exceeding the posterior border of eye and the apex of the protibia not enlarged laterally. The present study reviews the extensive material held by the Institute of Zoology at the Chinese Academy of Sciences Beijing, the Natural History Museum London, and the Zoological Institute Saint Petersburg, but also from some private collections. Previously published keys to the species of *Pseudocneorhinus* included those to the Korean (Han et al. 2000) and Japanese faunas (Morimoto et al. 2015), but no key to all the species has been published since Marshall’s (1934) review of the genus, in which the number of recognised species is half that recognised in the current paper; a full key is given below. In addition, illustrations of diagnostically important internal structures are provided.

## Materials and methods

Body length was measured in profile from the anterior margin of the eyes to the apex of the elytra, excluding the rostrum. All other measurements were taken in dorsal view: rostral length between anterior margins of eyes and anterior margin of epistome, rostral width as maximum width, pronotal and elytral length along midline, and their widths as maximum extension across. Dissected female genitalia were embedded in Solakryl BMX. Dried male genitalia were glued on the same mounting card as the insect. The terminology for rostrum and terminalia follows Oberprieler et al. (2014).

Photos of adults were taken with a Canon EOS 7D digital camera with an MP-E 65 mm macro lens and combined using CombineZP software. All habitus photos were edited with Adobe Photoshop CS3. Line drawings were made using a camera Lucida mounted on a Rathenow microscope. Maps were prepared with Simplemappr (Shorthouse 2010).

Available types of species hitherto described were studied, and lectotypes were selected when necessary according to Article 74.7.3 of the Code of Zoological Nomenclature.

Acronyms for depositories of the material are as follows:

**BMNH**The Natural History Museum, London, United Kingdom [formerly British Museum (Natural History)];

**CGTS** Christoph Germann collection, Rubigen, Switzerland;

**GOVI** Giuseppe Osella collection, Verona, Italy;

**IZCAS**Institute of Zoology, Chinese Academy of Sciences;

**JSPC** Jiří Skuhrovec collection, Praha, Czech Republic;

**MKBC** Michael Košťál collection, Brno, Czech Republic;

**MMTI** Massimo Meregalli collection, Torino, Italy;

**NHRS**Naturhistoriska Riksmuseet, Stockholm, Sweden;

**NMPC**Národní muzeum, Prague, Czech Republic;

**PBSP** Piotr Białooki, Sopot, Poland;

**PKSC** Petr Kresl collection, Spůle, Czech Republic;

**RBSC** Roman Borovec collection, Sloupno, Czech Republic;

**SMTD** Senckenberg Naturhistorische Sammlungen Dresden, Germany;

**SMNS**Staatliches Museum für Naturkunde, Stuttgart, Germany;

**UMO** University Museum, Oxford, United Kingdom;

**ZFMK**Zoologisches Forschungsmuseum Alexander Koenig, Bonn, Germany;

**ZIN**Zoological Institute of the USSR Academy of Sciences, Saint Petersburg, Russia.

## Taxonomy

### *Pseudocneorhinus* Roelofs, 1873

*Pseudocneorhinus* Roelofs, 1873: 177 (original description).

*Pseudocneorhinus*: Alonso-Zarazaga and Lyal 1999: 183 (catalogue); Han et al. 2000: 33 (Korean fauna); Borovec 2003: 31 (note); Borovec 2009: 76 (redescription of genus); Morimoto et al. 2015: 322 (Japanese fauna); Alonso-Zarazaga et al. 2017: 403 (catalogue).

#### Pseudocneorhinus angustus sp. nov.

Taxon classificationAnimaliaColeopteraCurculionidae

http://zoobank.org/61DDA9DF-3031-4340-812E-8135D54416C7

Figs 1 , 2 , 31

##### Type locality.

Valley of Fubianhe river (China: Sichuan).

##### Material examined.

**Holotype.** CHINA – **Sichuan Prov.** ♂; valley of Fubianhe river; 2 Aug. 1893; Potanin leg.; ZIN. **Paratype.** CHINA – **Sichuan Prov.** 1 ♂; same data as for holotype; 5 Aug. 1893; ZIN.

##### Description.

Body length: Holotype 3.41 mm, paratype 3.50 mm.

**Figures 1–8.**
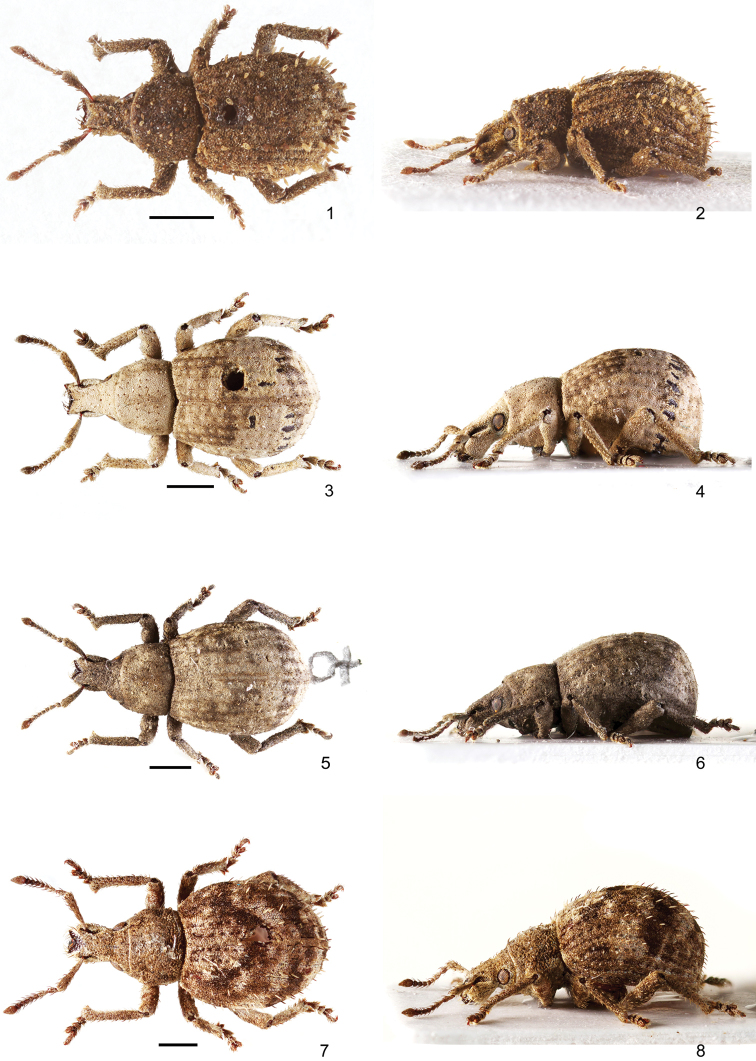
Habitus of *Pseudocneorhinus* species **1, 2***Pseudocneorhinusangustus* sp. nov., male, paratype, dorsal and lateral view **3, 4***P.glaber*, sp. nov., male, holotype, dorsal and lateral view **5, 6***P.glaber* sp. nov., female, paratype, dorsal and lateral view **7, 8***P.hlavaci* sp. nov., female, holotype, dorsal and lateral view. Scale bars: 1 mm.

*Body* (Figs 1, 2) blackish, basal half of antennal scape, funicle and tarsi reddish brown, mucro and claws reddish. Appressed scales covering antennae, head, pronotum, elytra and legs, except antennal club; scales on elytra small, irregularly angular, with indistinct depression in the middle; 4–6 scales across interval width, somewhat sparse, narrowly separate; scales light brownish with V-shaped transverse stripe from greyish scales on elytral declivity. Raised elytral setae conspicuous, erect, long and wide, spatulate, positioned in single dense row only on odd intervals, with short longitudinal clumps of intervals 3 and 5 on declivity; setae white greyish and blackish, alternating irregularly. Semierect setae on pronotum, head and rostrum half as long and wide as elytral ones, irregularly scattered. Antennae and legs except for basal half of scape with semierect moderately long setae, prominent from outline.

*Rostrum* (Figs 1, 2) long and slender, 1.09–1.11 × as long as wide, abruptly widened from base to basal one-fourth, then weakly tapered anteriad, with straight sides. Epifrons somewhat tapered from base to apex, with straight sides, longitudinally depressed along the whole length. Epistome short and wide, apices distinctly wider than anterior part of epifrons, separated from frons by indistinct slender carina. Frons squamose with three pairs of stout, yellowish setae. Antennal scrobe in dorsal view almost invisible; in lateral view curved, short, directed towards eyes. Rostrum in lateral view strongly convex, indistinctly separated from head by shallow transverse depression. Eyes hardly prominent from outline of head.

*Antennae* slender with robust scape. Scape as long as funicle, weakly curved, regularly but distinctly enlarged apicad in apical half, at apex 1.4–1.6 × as wide as club. Funicle segment 1 as long as and slightly wider than segment 2, both conical; segment 1 twice as long as wide; segment 2 2.3–2.4 × as long as wide; segments 3 and 4 1.1–1.2 × as long as wide; segments 5 and 6 isodiametric; segment 7 1.1–1.2 × as wide as long; club 1.7–1.8 × as long as wide.

*Pronotum* (Figs 1, 2) 1.26–1.27 × as wide as long, with weakly rounded sides, widest at midlength, more tapered anteriad than posteriad. Disc regularly convex. Anterior border in lateral view sinuose, ocular lobes well developed. Base weakly convex.

*Elytra* (Figs 1, 2) slender, elongate oval, 1.25–1.29 × as long as wide, widest behind midlength, not wider at shoulders. Striae distinct, weakly curved on elytral disc. Even intervals wider and more elevated than odd intervals, mainly in basal part and on elytral declivity. Base straight laterally, sinuate only in middle between third intervals. Elytra in lateral view moderately convex.

*Protibiae* rounded at apex, with fringe of very short yellowish setae, mucronate. Inner side of all tibiae without teeth. Metatibial corbels squamose. Tarsi robust; segment 2 1.2–1.3 × as wide as long; segment 3 1.3–1.4 × as wide as long and 1.3–1.4 × as wide as segment 2; onychium 1.1–1.2 × as long as segment 3. Claws fused in basal half.

*Penis* (Fig. 31) short with weakly rounded sides; apex distinctly tapered, subtriangular with concave sides. Penis in lateral view short and wide, obtuse with slender elongated apex in ventral side.

*Female genitalia* unknown.

##### Biology.

Unknown.

##### Distribution.

China: Sichuan (Fig. 52).

##### Etymology.

The name is a Latin adjective meaning narrow and used to refer to the unusually slender elytra.

##### Differential diagnosis.

*Pseudocneorhinusangustus* is similar to *P.hirsutus* (Formánek, 1916) and *P.squamosus* Marshall, 1934 in having distinctly enlarged antennal scapes, squamose frons, only medially (between third intervals) sinuate elytral base and small body size. It is possible to distinguish it from both these species by raised setae confined to odd intervals (*P.hirsutus* and *P.squamosus* have setae on all intervals), rostrum 1.1 × longer than wide (*P.hirsutus* and *P.squamosus* have rostrum isodiametric), epifrons tapered apicad with straight sides (*P.hirsutus* and *P.squamosus* have epifrons parallel-sided, at base weakly concave), epistome with apices distinctly wider than anterior part of epifrons (*P.hirsutus* and *P.squamosus* have epistome with apices distinctly narrower than anterior part of epifrons) and elytra slender, interval 1 at declivity much wider than on the disc (*P.hirsutus* and *P.squamosus* have elytra wider with interval 1 equally wide along the whole length). Other similar species with raised setae only on odd intervals are *P.alternans* Marshall, 1934, *P.setosicallus* sp. nov. and *P.subcallosus* (Voss, 1956). *P.angustus* can be distinguished from all these three species by smaller body size, long and wide spatulate raised elytral setae and apically distinctly enlarged scapes (apex wider than club). *Pseudocneorhinusangustus* is most similar to *P.setosicallus* because of long erect setae on the elytra; they can be distinguished by the characters specified in the key below.

#### Pseudocneorhinus glaber sp. nov.

Taxon classificationAnimaliaColeopteraCurculionidae

http://zoobank.org/C3880FA1-F1EA-4050-835D-9588C617CA3D

Figs 3–6 , 32 , 37

##### Type locality.

Anji County, Longwang Mountain (China: Zhejiang).

##### Material examined.

**Holotype.** CHINA – **Zhejiang Prov.** ♂; Anji, Longwangshan [安吉龙王山]; 450 m a.s.l.; 16 May 1996; H. Wu leg. [吴鸿]; IZCAS, IOZ(E)1965363.

**Paratypes.** CHINA – **Zhejiang Prov.** 2 ♂♂; same data as for holotype; IZCAS, IOZ(E)1965355, IOZ(E)1965364; 1 ♀; same data as for holotype; IZCAS, IOZ(E)1786461; 1 ♂; same data as for holotype; 600 m a.s.l.; 13 Jun. 1996; W.Z. Li leg. [李文柱]; IZCAS, IOZ(E)1965354; 1 ♂; same data as for holotype; 11 Jun. 1996; W.Z. Li leg. [李文柱]; IZCAS, IOZ(E)1965353; 14 ♂♂; Lin’an, West Tianmushan, Longwangshan Gang [临安西天目山龙王山岗]; 30°13.027'N, 119°24.929'E; 1452 m a.s.l.; 25 Jul. 2011; N. Yang leg. [杨妮]; IZCAS, IOZ(E)1965337–1965345, IOZ(E)1965347–1965349, IOZ(E)1965351, IOZ(E)1965362; 4 ♀♀; same data as for preceding; IZCAS, IOZ(E)1965346, IOZ(E)1965350, IOZ(E)1965352, IOZ(E)1965361; 1 ♂; Lin’an, West Tianmushan, skyline drive [临安西天目山盘山公路]; 600 m a.s.l.; 26 Jul. 2011; N. Yang leg. [杨妮]; IZCAS, IOZ(E)1965336; 1 ♂; Lin’an, West Tianmushan, Xiaoshilin [临安西天目山小石林]; 1450 m a.s.l.; 30 Jul. 2011; N. Yang leg. [杨妮]; IZCAS, IOZ(E)1965335; 1 ♂; West Tianmushan, Xianrending [西天目山仙人顶]; 1500 m a.s.l.; 6 Jun. 1998; H. Wu leg. [吴鸿]; IZCAS, IOZ(E)1798071; 1 ♀; West Tianmushan, Sanliting [西天目山三里亭]; 25 Aug. 1998; H. Wu leg. [吴鸿]; IZCAS, IOZ(E)1798081; 1 ♀; West Tianmushan, Kaishan Laodian [西天目山开山老殿]; 1050 m a.s.l.; 23 Jun. 1998; H. Wu leg. [吴鸿]; IZCAS, IOZ(E)1798094; 1 ♂; West Tianmushan, Sanmuping [西天目山三亩坪]; 30 Jul. 1998; H. Wu leg. [吴鸿]; IZCAS, IOZ(E)1798099; 3 ♂♂; Lin’an City, Qingliangfeng county, Shunxi village [临安市清凉峰镇顺溪村]; 30°03.041'N, 118°56.550'E; 400 m a.s.l.; 9 Aug. 2008; J. Yang leg. [杨娟]; IZCAS, IOZ(E)1965311, IOZ(E)1965312, IOZ(E)1965356; 2 ♂♂; same data as for preceding; 10 Aug. 2008; IZCAS, IOZ(E)1965313, IOZ(E)1965314; 1 ♀; same data as for preceding; IOZ(E)1965357; 2 ♂♂; West Qianqingtang [西千顷塘]; 30°18.023'N, 119°07.037'E; 1140 m a.s.l.; 6 Aug. 2008; J. Yang leg. [杨娟]; beat sheet [振布]; IZCAS, IOZ(E)1965315, IOZ(E)1965320; 2 ♀♀; same data as for preceding; IZCAS, IOZ(E)1965358, IOZ(E)1965360; 9 ♂♂; same data as for preceding; 7 Aug. 2008; IZCAS, IOZ(E)1965316, IOZ(E)1965321–1965323, IOZ(E)1965326, IOZ(E)1965329, IOZ(E)1965330, IOZ(E)1965333, IOZ(E)1965359; 10 ♀♀; same data as for preceding; IZCAS, IOZ(E)1965317–1965319, IOZ(E)1965324, IOZ(E)1965325, IOZ(E)1965327, IOZ(E)1965328, IOZ(E)1965331, IOZ(E)1965332, IOZ(E)1965334.

##### Description.

Body length: 4.63–5.19 mm, holotype 4.75 mm.

*Body* (Figs 3–6) blackish, mucro and fringe of setae on protibia yellowish to reddish, claws brownish. Appressed scales covering antennae, head, pronotum, elytra and legs, except antennal club; scales on dorsal part of body small, irregularly angular, depressed in the middle, 8–9 scales across elytral interval width, narrow separate; scales light greyish with feeble pearly sheen, on elytra with slender transverse dark brownish stripe at anterior third and wider dark brownish stripe at apical third. Semiappressed elytral setae inconspicuous, strongly inclined, piliform to bristle-shaped, about as long as half of width of elytral interval, visible only in apical part or at base of elytra. Pronotum and head capsule and rostrum with identical semiappressed setae, these sparse and irregularly scattered, on pronotum directed transversely. Antennae and legs except for basal half of scape with semierect moderately long setae, prominent from outline.

*Rostrum* (Figs 3–6) in males longer and more slender than in females, in males 1.17–1.20 × as long as wide, in females 1.04–1.07 × as long as wide, regularly enlarged from base to midlength, then tapered anteriad with regularly rounded sides. Epifrons tapering from base to midlength and widened again with slightly rounded sides at basal and apical half, at apex narrower than at base, longitudinally widely and shallowly depressed. Epistome V-shaped, long, conspicuous, separated by slender carina from frons, in females at apex narrower than epifrons at apex, in males lengthened and curved along anterior border of rostrum, wider than epifrons at apex. Frons as a very narrow glabrous strip along epistome, bearing four pairs of stout and long apical setae, obliquely directed anteriad. Scrobe in doral view invisible; in lateral view narrow, subparallel-sided, long, weakly curved, directed towards middle of eyes. Rostrum in lateral view somewhat convex, in males longer and more slender than in females, separated from head by shallow transverse depression. Eyes hardly prominent from outline of head.

*Antennae* slender, funicle 1.2 × as long as scape. Scape slender, gradually and regularly enlarged apicad, at apex as wide as club. Funicle segments 1 and 2 conical, long, funicle segment 1 slightly longer and wider than segment 2, the both 1.6–1.7 × as long as wide; segments 3 and 4 1.1 × as long as wide; segments 5 and 6 isodiametric; segment 7 1.1–1.2 × as long as wide.

*Pronotum* (Figs 3–6) 1.26–1.31 × as wide as long, widest at basal third, with distinctly rounded sides, more strongly tapered anteriad than posteriad. Disc regularly convex. Base weakly convex. Pronotum in lateral view moderately convex, ocular lobes well developed.

*Elytra* (Figs 3–6) 1.21–1.31 × as long as wide, ovoid, widest at apical third; shoulders absent, elytra at base hardly wider than base of pronotum, behind base with straight to slightly concave sides. Striae wide and distinct, punctate, punctures wide and completely hidden by appressed scales; intervals weakly convex, odd intervals slightly more so than even ones, equally wide, weakly wider than striae. Elytra in lateral view distinctly convex.

*Protibiae* rounded at apex, with fringe of short and fine yellowish setae, mucronate, inner margin with 3–4 very small black, almost indistinct teeth. Metatibiae not denticulate; metatibial corbels densely squamose. Tarsi short, segment 2 1.4–1.5 × as wide as long; segment 3 1.4–1.5× as wide as long and 1.4–1.5 × as wide as segment 2; onychium 0.7–0.8 × as long as segment 3. Claws solidly fused in basal half, almost parallel-sided in apical half.

*Penis* (Fig. 32) short and wide, in ventral view slightly and regularly enlarged apicad, with straight sides, apex triangular with small triangular ends on sides; in lateral view almost straight, distinctly enlarged apicad, apex slender, elongate, dorsal border lengthened, lobe-like.

*Female genitalia*. Sternite VIII umbrella-shaped with short apodeme. Gonocoxites flat, subtriangular, with long apical styli, laterally prominent, armed with setae. Spermatheca (Fig. 37) with cornu long and regularly curved; corpus enlarged oval, ramus and nodulus not differentiated.

##### Biology.

Unknown.

##### Distribution.

China: Zhejiang (Fig. 52).

##### Etymology.

The Latin name, meaning smooth and without setae, refers to body with barely visible, inconspicuous, short piliform semi-appressed setae.

##### Differential diagnosis.

*Pseudocneorhinusglaber* has inconspicuous elytral vestiture consisting of short, piliform setae that are semi-appressed and barely visible at apex and base in lateral view; all other species have elytra with conspicuous, moderate to very long setae of various widths and shapes, which are always more or less erect and well visible even in dorsal view. *Pseudocneorhinusglaber* resembles also species of the genus *Rhinodontodes* in having a long rostrum and medially constricted epifrons, but the epistome does not exceed the outline of the rostrum and the protibiae are straight.

#### Pseudocneorhinus hlavaci sp. nov.

Taxon classificationAnimaliaColeopteraCurculionidae

http://zoobank.org/AD7ABB15-DC0A-4A7C-8D41-B7A0A247E8FE

Figs 7 , 8 , 38

##### Type locality.

Dongling Mountains, Xiaolongmen, Liu Lang Yu (China: Beijing).

##### Material examined.

**Holotype.** CHINA – **Beijing** ♀; Dongling Mountains, Xiaolongmen, Liu Lang Yu; 39°58.2'N, 115°25.8'E; 1400 m a.s.l.; 15 Jun. 2001; J. Cooter & P. Hlaváč leg.; Litter; BMNH.

**Paratypes.** CHINA – **Beijing** 1 ♀; same data as for holotype; BMNH; 1 ♀; Xiaolongmen forestry station, Nan’gou [小龙门林场南沟]; 1140 m a.s.l.; 30 May–2 Jun. 2001; X.D. Yu leg. [于晓东]; *Larix* forest, pitfall trap [落叶松林, 杯诱]; IZCAS, IOZ(E)1965213; 2 ♀♀; Mentougou, Xiaolongmen [门头沟小龙门]; 39°57.6'N, 115°25.8'E; 1164–1210 m a.s.l.; 5 Jul. 2011; K.Y. Zhang leg. [张魁艳]; IZCAS, IOZ(E)1965297, IOZ(E)1965301; 1 ♀; same data as for preceding; G.X. Qiao & J. Chen leg. [乔格侠, 陈军]; IZCAS, IOZ(E)1965300; 1 ♀; Xiaolongmen forestry station, Nan’gou [小龙门林场南沟]; 1140 m a.s.l.; 18–21 Jul. 1999; X.D. Yu leg. [于晓东]; *Larix* forest, pitfall trap [落叶松林, 杯诱]; IZCAS, IOZ(E)1965194; 1 ♀; Xiaolongmen [小龙门]; 1200–1350 m a.s.l.; 19 Aug. 1999; W.P. Xie leg. [谢为平]; IZCAS, IOZ(E)1965309; 1 ♀♀; Dongling Shan, 100 km W of Beijing; 1500 m a.s.l.; 12–15 Jun. 2000; Zd. Jindra leg.; NMPC; 3 ♀♀; same data as for preceding; RBSC; 1 ♀; same data as for preceding; UMO.

##### Description.

Body length: Holotype 5.25 mm, paratype 4.95–5.56 mm.

*Body* (Figs 7, 8) blackish, only antennal club and basal part of first tarsal segment, mucro, fringe of setae at apex of protibia, and claws reddish. Appressed scales on body dense, hiding integument, oval, weakly imbricate, finely longitudinally striate, 5–6 scales across interval width; scales dark brownish with small light brownish spots irregularly scattered on elytra. Raised elytral setae semierect, slender, lanceolate, somewhat shorter than width of one elytral interval, with single sparse, regular row on each interval, setae greyish and blackish, alternating irregularly. Semierect setae on pronotum somewhat shorter than elytral ones, sparse, irregularly scattered. Semiappressed setae on head and rostrum half as long as pronotal setae. Antennae and legs except of basal half of scape with semierect moderately long setae, prominent in outline.

*Rostrum* (Figs 7, 8) long, 1.09–1.13 × as long as wide, regularly distinctly enlarged from base to antennal insertion, then rounded around apex, without abrupt widening at base. Epifrons tapered from base to midlength and widened again, at apex as wide as at base, longitudinally depressed, with somewhat swollen borders. Epistome V-shaped, long, conspicuous, separated by slender carina from frons, at apex as wide as epifrons in narrowest part. Frons glabrous, V-shaped, as a strip along epistome, bearing five pairs of stout, long apical setae, obliquely directed anteriad. Scrobe in dorsal view visible only in apical part as slender furrow; in lateral view narrow, short, curved, directed towards eye. Rostrum in lateral view somewhat convex, long and slender, separated from head by shallow transverse depression. Eyes weakly prominent from outline of head.

*Antennae* slender. Scape as long as funicle, straight, weakly and regularly enlarged to apex, at apex only slightly wider than club. Funicle segment 1 as long as and only slightly wider than segment 2, both conical; segment 1 1.7–1.8 × as long as wide; segment 2 1.9–2.0 × as long as wide; segment 3 1.2 × as long as wide; segment 4 1.1 × as long as wide; segments 5 and 6 isodiametric; segment 7 1.1 × as wide as long; club 1.6–1.7 × as long as wide.

*Pronotum* (Figs 7, 8) 1.42–1.47 × as wide as long, regularly convex on the disc, widest at midlength, with weakly rounded sides, more strongly tapered anteriad than posteriad. Base indistinctly convex, almost straight. Pronotum in lateral view convex, ocular lobes well developed.

*Elytra* (Figs 7, 8) regularly oval, 1.19–1.24 × as long as wide, widest at midlength; shoulders regularly rounded. Striae distinct; intervals almost flat, equally wide and convex. Elytra in lateral view distinctly convex.

*Protibiae* rounded at apex, with fringe of short and fine reddish setae, mucronate, on inner margin with 4–5 black, very small and indistinct teeth. Metatibiae with 1–3 black, very small and indistinct teeth in apical half; metatibial corbels squamose. Tarsi robust, segment 2 1.1–1.2 × as wide as long; segment 3 1.4–1.5 × as wide as long and 1.5–1.6 × as wide as segment 2; onychium equally long to 1.1 × as long as segment 3. Claws fused in basal half.

Male genitalia unknown.

*Female genitalia*. Sternite VIII umbrella-shaped with short apodeme. Gonocoxites flat, weakly sclerotised with short apical styli, armed with setae. Spermatheca (Fig. 38) with cornu long and regularly curved; ramus short twice as wide as long; nodulus short as ramus, half as wide as ramus, returned.

##### Biology.

The specimens were sifted from forest litter.

##### Distribution.

China: Beijing (Fig. 52).

##### Etymology.

The newly described species is dedicated to the collector, our friend Peter Hlaváč (Prague, Czech Republic), well-known specialist of Staphylinidae (Pselaphinae, Scydmaeninae) and also Curculionidae.

##### Differential diagnosis.

*Pseudocneorhinushlavaci* is most similar to *P.sellatus* Marshall, 1934 in terms of size, overall shape, regular intervals, and dorsal contour of rostrum (i.e., evenly enlarged apically, base not abruptly widened). It is easily distinguishable from it by elytral setae conspicuous and semierect, rostrum slightly longer than wide with straight sides in basal half and epifrons without longitudinal carina.

#### Pseudocneorhinus obliquehumeralis sp. nov.

Taxon classificationAnimaliaColeopteraCurculionidae

http://zoobank.org/A6963E03-622F-4F29-92ED-355101704F84

Figs 9 , 10

##### Type locality.

Xinglongshan b. Yuzhong, loc. Yangzhai (China: Gansu).

##### Material examined.

**Holotype.** CHINA – **Gansu Prov.** ♀; Xinglongshan b. Yuzhong, Yangzhai; 2500–3000 m a.s.l.; 22–26 Jul. 1993; Heinz leg.; SMNS.

**Paratype.** CHINA – **Shaanxi Prov.** 1 ♀; Qing Ling Shan mts., road Baoji – Taibai vill., Pass 40 km S Baoji; 21–23 Jul. 1998; Z. Jindra leg.; RBSC.

##### Description.

Body length: holotype 5.31 mm, paratype 5.44 mm.

**Figures 9–16.**
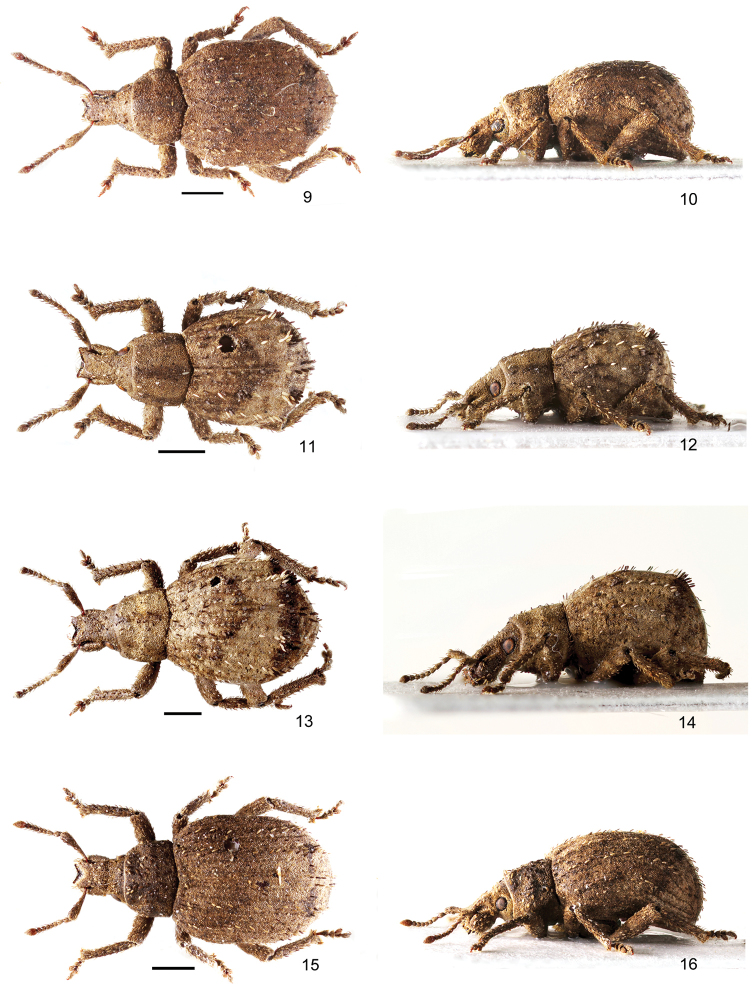
Habitus of *Pseudocneorhinus* species **9, 10***P.obliquehumeralis* sp. nov., female, paratype, dorsal and lateral view **11, 12***P.setosicallus* sp. nov., male, holotype, dorsal and lateral view **13, 14***P.setosicallus* sp. nov., female, paratype, dorsal and lateral view **15, 16***P.alternans*, female, dorsal and lateral view. Scale bars: 1 mm.

*Body* (Figs 9, 10) blackish, only very short basal part of scape, club, mucro, and claws brownish and fringe of short setae at apex of protibiae yellowish. Appressed scales on body except pronotum, head capsule, rostrum and club imbricate, oval, small, finely longitudinally striate; 6–7 scales across interval width. Pronotum, head capsule and rostrum with appressed scales assembling on margins, irregularly tricuspid, narrowly separate. Club finely setose. Scales light brownish, elytra with narrow, transverse dark brownish stripe V-shaped at anterior third and straight at declivity. Elytra with narrow, subspatulate, longitudinally finely striate setae, appressed on disc, semiappressed on declivity, forming regular dense row on each odd interval, and very sparse, hardly visible row on even intervals, about as long as half width of one interval, light grey brownish, on even intervals dark brownish. Pronotum, head capsule and rostrum with identical appressed setae, on pronotum orientated transversely, on rostrum longitudinally, sparsely irregularly scattered. Scape and femora with moderately long semierect setae; funicle, tibiae. and tarsi with identical semierect setae, prominent from outline.

*Rostrum* (Figs 9, 10) short and wide, 1.02–1.04 × as wide as long, narrowest at base, regularly moderately enlarged apicad with almost straight sides. Epifrons in basal almost two thirds tapered anteriad, in anterior third slightly enlarged again, in both parts with weakly convex sides, longitudinally shallowly depressed. Epistome V-shaped, moderately sized, separated by slender carina from frons, at apex distinctly narrower than apical part of epifrons. Frons as very slender glabrous strip along epistome, bearing 3–4 pairs of long, stout setae, obliquely directed anteriorly. Scrobe in dorsal view visible as very slender furrow in apical part; in lateral view short, weakly curved, narrow, directed towards middle of eyes. Rostrum in lateral view weakly convex, separated from head by shallow transverse depression. Eyes weakly prominent from outline of head.

*Antennae* slender; scape slender, weakly curved, regularly enlarged apicad, at apex same width as club. Funicle 1.2–1.3 × as long as scape; funicle segments 1 and 2 equally long, conical, segment 1 slightly wider than segment 2; segment 1 1.5–1.6 × as long as wide; segment 2 1.8–2.0 × as long as wide; segments 3 and 4 1.2–1.3 × as long as wide; segments 5 and 6 isodiametric, segment 7 1.1 × as wide as long.

*Pronotum* (Figs 9, 10) 1.53–1.58 × as wide as long, widest at midlength, with moderately rounded sides, distinctly more tapered anteriad than posteriad. Disc regularly convex. Base V-shaped. Pronotum in lateral view almost flat, ocular lobes weakly developed.

*Elytra* (Figs 9, 10) 1.27–1.33 × as long as wide, long-oval; shoulders angulate to base and to lateral margins, obliquely subtruncate; sides sub-parallel; apex broadly rounded. Striae punctate, punctures small, hidden by appressed scales. Stria 1 at base curved laterally, sutural interval at base enlarged. Odd intervals flat, wide; even intervals weakly elevated, intervals 3, 5 and 7 enlarged at declivity and with low but distinct longitudinal prominence, the biggest at interval 3. Base arched. Elytra in lateral view weakly convex.

*Protibiae* moderately slender, with straight lateral margin, rounded at apex, with fringe of short and fine yellowish setae, mucronate and not denticulate. Metatibiae with four very small, almost indistinct denticles at apical half; metatibial corbels densely squamous with two, equally long mucros, curved inside. Tarsi slender; segment 2 1.1–1.2 × as wide as long; segment 3 1.3–1.4 × as wide as long and 1.5–1.6 × as wide as previous segment; onychium 1.1 × as long as segment 3. Claws solidly fused in basal half, weakly separate in apical half.

Male genitalia unknown.

*Female genitalia*. Sternite VIII umbrella-shaped with short apodeme. Gonocoxites flat, moderately slender with long apical styli with setae, prominent laterally. Spermatheca not examined.

##### Biology.

Unknown.

##### Distribution.

China: Gansu, Shaanxi (Fig. 52).

##### Etymology.

The Latin name, meaning with oblique shoulders, refers to obliquely subtruncate shoulders, angled to elytral base and sides.

##### Differential diagnosis.

*Pseudocneorhinusobliquehumeralis* is similar to *P.alternans* by the following characters: oval elytra with distinct shoulders, slender antennal scapes, and raised elytral setae on odd intervals only. It can be distinguished from *P.alternans* by angular rather than regularly oblique shoulders, subdistally distinctly enlarged intervals 3 and 5 each with low longitudinal prominence, and more elongate funicular segments 3 and 4.

#### Pseudocneorhinus setosicallus sp. nov.

Taxon classificationAnimaliaColeopteraCurculionidae

http://zoobank.org/D09575D3-9616-43A2-8C66-E315CEA8F149

Figs 11–14 , 33 , 39

##### Type locality.

Wanxian County, Wang’erbao Natural Reserve (China: Chongqing).

##### Material examined.

**Holotype.** CHINA – **Chongqing** ♂; Wanxian county, Wang’erbao [万县王二包]; 1200 m a.s.l.; 27 May 1994; Y.W. Zhang leg. [章有为]; IZCAS, IOZ(E) 1786276.

**Paratypes.** CHINA – **Chongqing** 11 ♂♂; same data as for holotype; IZCAS, IOZ(E)1786279, IOZ(E)1786280, IOZ(E)1786282–1786286, IOZ(E)1786301, IOZ(E)1786302, IOZ(E)1786374, IOZ(E)1786375; 4 ♀♀; same data as for holotype; IZCAS, IOZ(E)1786278, IOZ(E)1786281, IOZ(E)1786287, IOZ(E)1786288; 2 ♂♂; same data as for holotype; J. Yao leg. [姚建]; IZCAS, IOZ(E)1786316, IOZ(E)1786317; 5 ♂♂; same data as for holotype; W.Z. Li leg. [李文柱]; IZCAS, IOZ(E)1786320, IOZ(E)1786321, IOZ(E)1786362–1786364; 3 ♀♀; same data as for preceding; IZCAS, IOZ(E)1786318, IOZ(E)1786319, IOZ(E)1786322; 2 ♂♂; same data as for holotype; X.K. Yang leg. [杨星科]; IZCAS, IOZ(E)1786334, IOZ(E)1786348; 3 ♀♀; same data as for preceding; IZCAS, IOZ(E)1786335, IOZ(E)1786349, IOZ(E)1786350; 12 ♂♂; same data as for preceding; 28 May 1994; IZCAS, IOZ(E)1786273, IOZ(E)1786274, IOZ(E)1786326, IOZ(E)1786327, IOZ(E)1786330, IOZ(E)1786331, IOZ(E)1786337–1786340, IOZ(E)1786342, IOZ(E)1786343; 8 ♀♀; same data as for preceding; IZCAS, IOZ(E)1786328, IOZ(E)1786329, IOZ(E)1786332, IOZ(E)1786333, IOZ(E)1786341, IOZ(E)1786345–1786347; 6 ♂♂; same data as for holotype; 28 May 1994; IZCAS, IOZ(E)1786304, IOZ(E)1786306, IOZ(E)1786307, IOZ(E)1786376–1786378; 5 ♀♀; same data as for preceding; IZCAS, IOZ(E)1786303, IOZ(E)1786305, IOZ(E)1786379–1786381; 2 ♂♂; same data as for preceding; W.Z. Li leg. [李文柱]; IZCAS, IOZ(E)1786324, IOZ(E)1965620; 1 ♀; same data as for preceding; IZCAS, IOZ(E)1786323; 1 ♀; same data as for preceding; J. Yao leg. [姚建]; IZCAS, IOZ(E)1786313; 2 ♀♀; same data as for holotype; 29 May 1994; W.Z. Li leg. [李文柱]; IZCAS, IOZ(E)1786365, IOZ(E)1786366; 1 ♂; same data as for preceding; J. Yao leg. [姚建]; IZCAS, IOZ(E)1786314; 1 ♂; same data as for preceding; 27 Sep. 1994; IZCAS, IOZ(E)1965239; 1 ♂; same data as for preceding; 1300 m a.s.l.; S.M. Song leg. [宋士美]; IZCAS, IOZ(E)1965257; 3 ♀♀; same data as for holotype; 28 Sep. 1994; J. Chen leg. [陈军]; IZCAS, IOZ(E)1965235, IOZ(E)1965236, IOZ(E)1965244; 2 ♂♂; same data as for holotype; 29 Sep. 1994; F.S. Li leg. [李法圣]; IZCAS, IOZ(E)1786372, IOZ(E)1786373; 1 ♂; same data as for preceding; J. Chen leg. [陈军]; IZCAS, IOZ(E)1965256; 1 ♂; same data as for holotype; 30 Sep. 1994; J. Yao leg. [姚建]; IZCAS, IOZ(E)1965254; 3 ♂♂; same data as for preceding; 1300 m a.s.l.; S.M. Song leg. [宋士美]; IZCAS, IOZ(E)1965237, IOZ(E)1965242, IOZ(E)1965262; 2 ♀♀; same data as for preceding; IZCAS, IOZ(E)1965258, IOZ(E)1965263; 7 ♂♂; same data as for holotype; 30 Sep. 1994; J. Chen leg. [陈军]; IZCAS, IOZ(E)1965232, IOZ(E)1965234, IOZ(E)1965240, IOZ(E)1965241, IOZ(E)1965243, IOZ(E)1965259, IOZ(E)1965261; 3 ♀♀; same data as for preceding; IZCAS, IOZ(E)1965233, IOZ(E)1965238, IOZ(E)1965255; 3 ♂♂; same data as for holotype; 22 May 1993; S.Y. Wang leg. [王書永]; IZCAS, IOZ(E)1786309, IOZ(E)1786367, IOZ(E)1786369; 2 ♀♀; same data as for preceding; IZCAS, IOZ(E)1786308, IOZ(E)1786368; 2 ♀♀; same data as for holotype; 10 Jul. 1993; R.Z. Huang leg. [黄润质]; IZCAS, IOZ(E)1786310, IOZ(E)1786311; 1 ♂; same data as for preceding; X.L. Chen leg. [陈小琳]; IZCAS, IOZ(E)1786371; 1 ♂; same data as for preceding; J. Yao leg. [姚建]; IZCAS, IOZ(E)1786315; 1 ♀; same data as for preceding; IZCAS, IOZ(E)1786312; 3 ♂♂; same data as for holotype; 13 Aug. 1993; X.K. Yang leg. [杨星科]; IZCAS, IOZ(E)1786275, IOZ(E)1786290, IOZ(E)1786299; 7 ♂♂; same data as for preceding; 14 Aug. 1993; IZCAS, IOZ(E)1786292, IOZ(E)1786293, IOZ(E)1786296, IOZ(E)1786298, IOZ(E)1786355, IOZ(E)1786358, IOZ(E)1786359; 12 ♀♀; same data as for preceding; IZCAS, IOZ(E)1786289, IOZ(E)1786294, IOZ(E)1786295, IOZ(E)1786300, IOZ(E)1786336, IOZ(E)1786344, IOZ(E)1786351–1786354, IOZ(E)1786356, IOZ(E)1786357; 1 ♂; same data as for holotype; 11 Jun. 1993; W.Z. Li leg. [李文柱]; IZCAS, IOZ(E)1786325; 1 ♂; same data as for holotype; 15 Aug. 1993; X.K. Yang leg. [杨星科]; IZCAS, IOZ(E)1786297; 2 ♀♀; same data as for preceding; IZCAS, IOZ(E)1786277, IOZ(E)1786291; 2 ♀♀; same data as for preceding; 1300 m a.s.l.; IZCAS, IOZ(E)1786360, IOZ(E)1786361; 1 ♂; same data as for preceding; B.W. Sun leg. [孙宝文]; IZCAS, IOZ(E)1786370. – **Sichuan Prov.** 1 ♀; Nanjiang; 21–23 May 2002; E. Kučera leg.; PBSP.

##### Description.

Body length: 4.19–5.75 mm, holotype 5.38 mm.

*Body* (Figs 11–14) blackish, only short basal part of scape, mucro, fringe of setae at apex of protibia, and claws brownish. Appressed scales on body dense, hiding integument, irregularly angular, small, 8–9 scales across interval width, with small depression in the middle, only narrowly separate. Scales light brownish, elytra in the middle with wide lighter transverse stripe, wider towards sides, elytral declivity with straight transverse dark brownish stripe. Elytra with conspicuous erect setae, longer than half of interval width, lanceolate, apically pointed, longitudinally finely striate, whitish and blackish, with one sparse row on each odd interval and only sporadic setae on even intervals. Setae denser on interval 1 on apical declivity, creating large and wide tuft of setae on prominence on elytral declivity on interval 3, consisting of 18–20 setae and smaller tuft on prominence on interval 5, consisting of 8–10 setae, anterior part of setae on prominence whitish, posterior part blackish. Semierect setae on pronotum and head with rostrum more slender and shorter than elytral setae, sparsely irregularly scattered. Antennae and legs except of basal half of scape with semierect moderately long setae, prominent from outline.

*Rostrum* (Figs 11–14) short and wide, in males slightly longer than in females, in males 1.03–1.06 × as long as wide, in females isodiametric, from base regularly enlarged to midlength, then tapered anteriad with rounded sides. Epifrons with concave sides, narrowest at midlength, at apex narrower than at base, longitudinally depressed, with somewhat swollen borders. Epistome V-shaped, long, conspicuous, separated by slender carina from frons, in females slightly narrower at apex than apical part of epifrons, in males at apex wider than apical part of epifrons. Frons creating very slender glabrous strip along epistome, bearing five pairs of long, stout setae, obliquely directed anteriorly. Scrobe in dorsal view visible only in apical part as very slender furrow; in lateral view narrow, long, weakly curved, directed towards middle of eyes. Rostrum in lateral view somewhat convex, separated from head by shallow transverse depression. Eyes weakly prominent from outline of head.

*Antennae* slender. Scapes slender, regularly enlarged in basal half, parallel-sided in apical half, at apex as wide as club. Funicle 1.2–1.3 × as long as scape; funicle segment 1 as long as and as wide as segment 2, each 1.8–1.9 × as long as wide; segments 3–6 1.1 × as long as wide; segment 7 isodiametric.

*Pronotum* (Figs 11–14) 1.18–1.26 × as wide as long, widest at midlength, in basal half subparallel-sided, weakly tapered anteriad, with rounded sides. Disc regularly convex. Base weakly convex. Pronotum in lateral view almost flat, ocular lobes well developed.

*Elytra* (Figs 11–14) 1.15–1.20 × as long as wide, ovoid in dorsal view, at base about as wide as base of pronotum, shoulders not developed; elytra distinctly enlarged posteriad, widest at apical third. Striae distinctly punctate, punctures wide, completely hidden by appressed scales. Even intervals almost flat, odd intervals convex, intervals 3 and 5 at elytral declivity enlarged, forming short longitudinal prominence, on interval 3 larger than on interval 5. Elytra in lateral view distinctly convex.

*Protibiae* rounded at apex, with fringe of short and fine yellow-brownish setae, mucronate, not denticulate, with straight lateral margin. Metatibiae not denticulate; metatibial corbels densely squamose. Tarsi short, segment 2 1.2–1.3 × as wide as long; segment 3 1.5–1.6 × as wide as long and 1.5–1.6 × as wide as segment 2; onychium 0.8–0.9 × as long as segment 3. Claws solidly fused at basal half, almost parallel-sided at apical half.

*Penis* (Fig. 33) short and wide, in ventral view subparallel-sided with weakly concave sides, base and apex about equally wide, apex truncate with triangular point at the middle; in lateral view short and very wide, slightly curved, equally wide along the whole length with slender, moderately long elongate apex.

*Female genitalia*. Sternite VIII with plate umbrella-shaped and with short apodeme. Gonocoxites flat, moderately slender with long apical styli with setae, prominent laterally. Spermatheca (Fig. 39) with cornu short and wide, almost straight, corpus large, rounded; ramus subtriangular, about as long as wide, nodulus small, hump-shaped.

##### Biology.

Unknown.

##### Distribution.

China: Chongqing, Sichuan (Fig. 52).

##### Etymology.

The Latin name, meaning with setae on prominence, refers to the conspicuous tuft of setae on prominence on the elytral declivity.

##### Differential diagnosis.

*Pseudocneorhinussetosicallus* is similar to *P.alternans* and *P.subcallosus* because of its large size and erect setae on odd intervals. From *P.alternans*, currently known only from females, this species is easily separated mainly by having ovoid elytra without shoulders, with the greatest width in the apical third, long erect elytral setae, conspicuous longitudinal subapical prominence on intervals 3 and 5 bearing very dense tufts of whitish and blackish setae and by slender pronotum. From *P.subcallosus*, a species with very similar body shape, *P.setosicallus* is distinguishable by its long, lanceolate erect elytral setae, clearly visible in dorsal as well as in lateral view, while *P.subcallosus* has elytral setae appressed, subspatulate, barely visible only in lateral view. The subapical tuft on interval 3 consists of at least 15 setae in *P.setosicallus* but at most 10 in *P.subcallosus*. As stated below in the key, *P.setosicallus* also has a longer rostrum and second funicular segment. From *P.angustus*, a generally smaller species with similar long, conspicuously erect elytral setae, *P.setosicallus* is distinguishable by shorter, in basal half enlarged rostrum, at mid-length more constricted epifrons, narrower pronotum and other characters given in the key.

#### Other *Pseudocneorhinus* species examined

##### Pseudocneorhinus adamsi

Taxon classificationAnimaliaColeopteraCurculionidae

Roelofs, 1879

Figs 34 , 40

Pseudocneorhinus adamsi Roelofs, 1879: liii (original description); Han et al. 2000: 34 (Korean fauna); Borovec 2009: 76 (check-list); Borovec 2013: 418 (catalogue); Morimoto et al. 2015: 338 (Japanese fauna); Alonso-Zarazaga et al. 2017: 403 (catalogue).

###### Material examined.

Other material. CHINA; 1 ♀; S. Manchuria, Chikuanshan; BMNH.

SOUTH KOREA; 25 ♂♂ ♀♀; Jinju, Witae, Sobae Mts.; 35°09.9'N, 127°49.4'E; 400 m a.s.l.; 16 May 2014; M. Košťál leg.; MKBC.

##### Pseudocneorhinus alternans

Taxon classificationAnimaliaColeopteraCurculionidae

Marshall, 1934

Figs 15 , 16 , 41

Pseudocneorhinus alternans Marshall, 1934: 7 (original description); Borovec 2009: 76 (check-list); Voss 1956: 24 (note); Borovec 2013: 418 (catalogue); Alonso-Zarazaga et al. 2017: 403 (catalogue).

###### Type material examined.

The original description was based on material from “China: S. Kansu, 1 ♀, 26.vi.1930, 1 ♀, 4.x.30 (Dr. Hummel)”. There is one specimen lacking head with rostrum in Marshall’s collection (BMNH) pinned on very slender and short paper label. This specimen is labelled as follows: Cotype [printed, circular label with yellow margin] / Kina S. Kansu [printed] / Sven Hedins Exp. Ctr. Asien Dr Hummel [printed] / Pres. by Imp. Inst. Ent. B. M. 1934-130. [printed] / 4/10 [handwritten] / Pseudocneorrhinus alternans Mshl. COTYPE ♀ [Marshall’s handwriting]. We have not designated the examined syntype specimen as lectotype in the view of the fact that it is incomplete and the species was described from two specimens.

###### Material examined.

Other material. CHINA – **Beijing** 1 ♀; Xiaolongmen forestry station, Nan’gou [小龙门林场南沟]; 1140 m a.s.l.; 25–28 Jun. 1999; X.D. Yu leg. [于晓东]; *Larix* forest, pitfall trap [落叶松林, 杯诱]; IZCAS, IOZ(E)1965163; 3 ♀♀; same data as for preceding; 30 May–2 Jun. 2001; IZCAS, IOZ(E)1965170–1965172; 2 ♀♀; Xiaolongmen forestry station, Nan’gou [小龙门林场南沟]; 1225 m a.s.l.; 18–21 Jul. 1999; X.D. Yu leg. [于晓东]; *Quercuswutaishanica* forest, pitfall trap [辽东栎林, 杯诱]; IZCAS, IOZ(E)1965165, IOZ(E)1965166; 1 ♀; Xiaolongmen [小龙门]; 1400 m a.s.l.; 14 Jun. 2001; S.Q. Ge leg. [葛斯琴]; IZCAS, IOZ(E)1965174; 1 ♀; North of Xiaolongmen forestry station [小龙门林场北]; 1190 m a.s.l.; 26–29 Jun. 1999; X.D. Yu leg. [于晓东]; *Pinustabulaeformis* forest, pitfall trap [油松林, 杯诱]; IZCAS, IOZ(E)1965178; 1 ♀; Xiaolongmen, Dongling Mountains; 39°58.2'N, 115°25.8'E; 1400 m a.s.l.; 15 Jun. 2001; J. Cooter & P. Hlaváč leg.; Liu Lang Yu Litter; BMNH; 3 ♀♀; Xiaolongmen; 39°58.074'N, 115°25.882'E; ca 1100 m a.s.l.; 9–13 Jun. 2004; J. Cooter leg.; BMNH; 1 ♀; Xialongmen, National Forest Reserve, 120 km W Beijing; 1100 m a.s.l.; 27 May 2005; M. Ritschard leg.; CGTS. – **Heilongjiang Prov.** 1 ♀; Qing Yuan, S Lang Xian, ca 30 km; 46°47.002'N, 129°04.349'E; 500–600 m a.s.l.; 26 May 2004; J. Cooter leg.; stream side; BMNH

##### Pseudocneorhinus bifasciatus

Taxon classificationAnimaliaColeopteraCurculionidae

Roelofs, 1880

**Figures 17–24.**
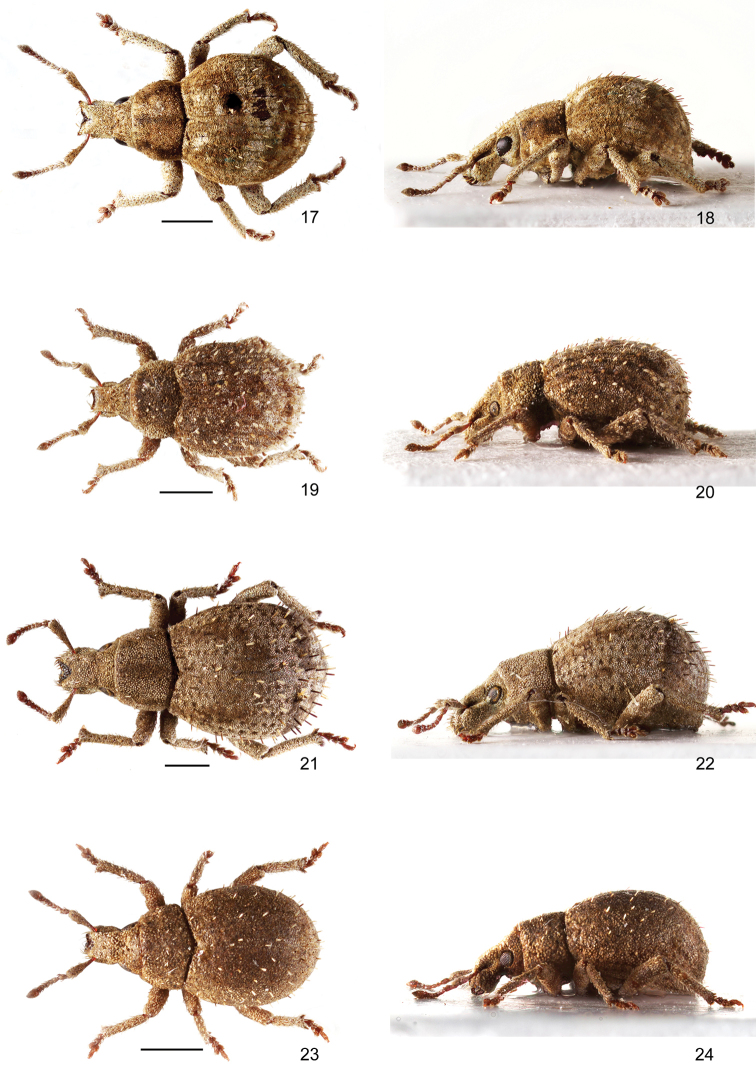
Habitus of *Pseudocneorhinus* species **17, 18***Pseudocneorhinusbifasciatus*, male, dorsal and lateral view **19, 20***P.hirsutus*, female, dorsal and lateral view **21, 22***P.longisetosus*, female, dorsal and lateral view **23, 24***P.minimus*, female, dorsal and lateral view. Scale bars: 1 mm.

Figs 17 , 18 , 35 , 42

Pseudocneorhinus bifasciatus Roelofs, 1880: 12 (original description); Marshall 1934: 10 (note); Voss 1956: 24 (note); Han et al. 2000: 35 (Korean fauna); Han and Yoon 2000: 259 (note); Borovec 2009: 76 (check-list); Borovec 2013: 418 (catalogue); Morimoto et al. 2015: 327 (Japanese fauna); Alonso-Zarazaga et al. 2017: 403 (catalogue).

###### Type material examined.

This species was described from “Plusieurs individus, par M. Lewis, du Japon”. We have studied one female specimen, 5.06 mm long, deposited in Marshall’s collection (BMNH), with the labels: Type [printed, circular label with red margin] / Japan G. Lewis 1910-320. [printed] / bifasciatus [handwritten].

###### Material examined.

Other material. CHINA – **Fujian Prov.** 1 ♀; Chong’an, Chengguan [崇安城关]; 240 m a.s.l.; 15 Jul. 1960; F.J. Pu leg. [蒲富基]; IZCAS, IOZ(E)1786483; 1 ♀; Chong’an, Xingcun, Tongmuguan [崇安星村桐木关]; 900 m a.s.l.; 10 Aug. 1960; Y. Zuo leg. [左永]; IZCAS, IOZ(E)1788386; 1 ♂; Jianyang, Huangkeng, Aotou [建阳黄坑坳头]; 950 m a.s.l.; 3 Jul. 1965; IZCAS, IOZ(E)1786480; 1 ♂; Jianyang, Huangkeng, Dazhulan [建阳黄坑大竹欄]; 900–1100 m a.s.l.; 7 May 1960; S.Q. Jiang leg. [姜勝巧]; IZCAS, IOZ(E)1788387; 1 ♀; Jianyang, Dazhulan [建阳大竹岚]; 4 Jul. 1965; IZCAS, IOZ(E)1788426; 1 ♂; Wuyi [武夷]; 27 Jun. 1982; K.C. Zhang leg. [张可池]; IZCAS, IOZ(E)1965245; 2 ♀♀; Jiangle, Longxishan [将乐龙栖山]; 800 m a.s.l.; 6 Aug. 1991; X.C. Zhang leg. [张晓春]; IZCAS, IOZ(E)17886463, IOZ(E)17886464; 1 ♂; Jiangle, Longxishan [将乐龙栖山]; 14 May 1991; R.Z. Zhang leg. [张润志]; IZCAS, IOZ(E)17886462; 3 ♀♀; Shaowu, Wushi [邵武乌石]; 6 Jun. 1965; IZCAS, IOZ(E)1786187, IOZ(E)1786189, IOZ(E)1786190; 1 spec.; Shaowu, Tachuland; 20 Jun. 1942; T. C. Maa leg.; BMNH; 6 ♀♀; Kuatun; Jun. 1946; Tschung Sen leg.; RBSC. – **Jiangsu Prov.** 1 spec.; Soochow; 18 Aug.1965; P. M. Hammond leg.; BMNH. – **Zhejiang Prov.** 13 spec.; Chusan is.; J. J. Walker leg.; BMNH; 1 spec.; Chusan, Pwanche; BMNH.

JAPAN; 5 spec.; G. Lewis leg.; BMNH.

##### Pseudocneorhinus hirsutus

Taxon classificationAnimaliaColeopteraCurculionidae

(Formánek, 1916)

Figs 19 , 20 , 43

Rhinodontus hirsutus Formánek, 1916: 33 (original description).
Pseudocneorhinus hirsutus : Marshall 1934: 8 (note); Borovec 2003: 49 (note); Borovec 2013: 418 (catalogue); Alonso-Zarazaga et al. 2017: 403 (catalogue).

###### Material examined.

Other material. CHINA – **Qinghai Prov.** 3 ♀♀; TIBET, Kuku-Nor; 3200 m a.s.l.; 1898; Hauser leg.; GOVI.

##### Pseudocneorhinus longisetosus

Taxon classificationAnimaliaColeopteraCurculionidae

Morimoto, 2015

Figs 21 , 22 , 44

Pseudocneorhinus longisetosus Morimoto, 2015: 339 (original description); Alonso-Zarazaga et al. 2017: 403 (catalogue).

###### Material examined.

Other material. RUSSIA; 8 ♀♀; Siberia or. mer., Primorje, Sichote – Alin Mts, Sokolči; 1–15 Jul.1990; S. Kadlec & J. Voříšek leg.; JSPC, RBSC; 19 ♀♀; Siberia or. Mer., Primorje, Ussuri res.; 20 Jul. 1990; S. Kadlec leg.; RBSC; 1 ♀; Siberia or. mer., Primorje, Komarovka flum, Kamenushka env., 300 m a.s.l.; May 1992; Voříšek leg.; RBSC; 1 ♀; Siberia or. mer., Kamenushka at Ussuriysk; 2 Aug.1992; J. Sawoniewicz leg.; RBSC; 1 ♀; Siberia or., Chechcir chrebet; 7 Jul.1977; Gottwald leg.; RBSC; 4 ♀♀; USSR, Chabarovsk; 7 Jul. 1981; Mejzlík leg.; RBSC; 1 ♀; Khabarovsk; 4 Jul.1977; Rataj leg.; MMTI.

##### Pseudocneorhinus minimus

Taxon classificationAnimaliaColeopteraCurculionidae

Roelofs, 1879

Figs 23 , 24 , 45

Pseudocneorhinus minimus Roelofs, 1879: liii (original description); Marshall 1934: 7 (note); Han et al. 2000: 36 (Korean fauna); Borovec 2009: 76 (check-list); Borovec 2013: 418 (catalogue); Morimoto et al. 2015: 333 (Japanese fauna); Alonso-Zarazaga et al. 2017: 403 (catalogue).

###### Type material examined.

This species was described from an unspecified number of specimens from “Japon”. We have studied one probably female specimen, well preserved and 2.94 mm long, deposited in Marshall’s collection (BMNH), labeled as follows: Type [printed, circular label with red margin] / Japan G. Lewis 1910-320. [printed] / minimus [handwritten].

###### Material examined.

Other material. CHINA – **Fujian Prov.** 1 ♀; Fenanina env., NW slope of Yunwu Shan; 1200 m a.s.l.; 3 Jun. 2000; Z. Jindra leg.; PKSC.

JAPAN; 3 ♀♀; G. Lewis leg.; BMNH.

##### Pseudocneorhinus obesus

Taxon classificationAnimaliaColeopteraCurculionidae

Roelofs, 1873

Figs 25 , 26 , 46

Pseudocneorhinus obesus Roelofs, 1873: 177 (original description); Marshall 1934: 9 (note); Voss 1956: 24 (note); Alonso-Zarazaga and Lyal 1999: 183 (catalogue); Han et al. 2000: 34 (Korean fauna); Borovec 2009: 76 (check-list); Borovec 2013: 418 (catalogue); Morimoto et al. 2015: 331 (Japanese fauna); Alonso-Zarazaga et al. 2017: 403 (catalogue).

###### Type material examined.

This species was described from “Quelques individus. Nagasaki”. There is one well preserved, 5.25 mm long, probably female specimen in Marshall’s collection (BMNH) below the name *Pseudocneorhinusobesus*, labeled as follows: Type H. T. [printed, circular label with red margin] / Japan G. Lewis 1910-320. [printed].

###### Material examined.

Other material. JAPAN; 2 ♀♀; G. Lewis leg.; BMNH; 1 ♀; Honshu, Akira Mt., Fyokai-San; 1 Jun. 1972; Takizava leg.; MMTI.

##### Pseudocneorhinus sellatus

Taxon classificationAnimaliaColeopteraCurculionidae

Marshall, 1934

Figs 27 , 28 , 47

Pseudocneorhinus sellatus Marshall, 1934: 8 (original description); Borovec 2009: 76 (check-list); Borovec 2013: 418 (catalogue); Alonso-Zarazaga et al. 2017: 403 (catalogue).

###### Material examined.

Other material. CHINA – **Beijing**; 2 ♀♀; Mentougou, Xiaolongmen [门头沟小龙门]; 39°57.6'N, 115°25.8'E; 1164–1210 m a.s.l.; 5 Jul. 2011; G.X. Qiao & J. Chen leg. [乔格侠, 陈军]; IZCAS, IOZ(E)1965155, IOZ(E)1965156; 1 ♀; same data as for preceding; K.Y. Zhang leg. [张魁艳]; IZCAS, IOZ(E)1965154; 1 ♀; Xiaolongmen forestry station, Nan’gou [小龙门林场南沟]; 19 Jul. 1999; T.H. Luo leg. [罗天宏]; heap of grass trap [堆诱]; IZCAS, IOZ(E)1965153; 1 ♀; North of Xiaolongmen forestry station [小龙门林场北]; 1190 m a.s.l.; 26–29 Jun. 1999; X.D. Yu leg. [于晓东]; *Pinustabulaeformis* forest, pitfall trap [油松林, 杯诱]; IZCAS, IOZ(E)1965152; 2 ♀♀; Xiaolongmen [小龙门]; 20 Jul. 1999; H.Z. Zhou leg. [周红章]; IZCAS, IOZ(E)1965150, IOZ(E)1965151; 1 ♀; Xiaolongmen, Dongling Mts.; 39°58.2'N, 115°25.8'E; 1450 m a.s.l.; 13 Jun. 2001; J. Cooter leg.; BMNH; 2 ♀♀; Xiaolongmen, Dongling Mts., Liu Lang Yu; 39°58.2'N, 115°25.8'E; 1400 m a.s.l.; 6 Jun. 2001; J. Cooter leg.; BMNH; 18 ♀♀; Dongling Shan, 100 km W of Beijing; 1500 m a.s.l.; 12–15 Jun. 2000; Z. Jindra leg.; NMPC, PKSC, RBSC. – **Sichuan Prov.**; 1 ♀; Nanping, Juizhaigou; 7–12 Jun. 2009; E. Kučera leg.; RBSC.

##### Pseudocneorhinus setosus

Taxon classificationAnimaliaColeopteraCurculionidae

Roelofs, 1879

Fig. 48

Pseudocneorhinus setosus Roelofs, 1879: liii (original description); Marshall 1934: 9 (note); Voss 1956: 24 (note); Han et al. 2000: 35 (Korean fauna); Borovec 2009: 76 (check-list); Borovec 2013: 418 (catalogue); Morimoto et al. 2015: 334 (Japanese fauna); Alonso-Zarazaga et al. 2017: 403 (catalogue).

###### Type material examined.

This species was described from an unspecified number of specimens from “Japon”. We have studied one probably female specimen, well preserved and 4.88 mm long, deposited in Marshall’s collection (BMNH), with the labels: Type [printed, circular label with red margin] / Japan G. Lewis 1910-320. [printed] / Lewis [handwritten] / Pseudocn. setosus R. Japon L. [handwritten].

###### Material examined.

Other material. CHINA – **Fujian Prov.** 1 ♀; Kuatun; Jun. 1946; Tschung Sen leg.; RBSC.

JAPAN; 1 ♀; Nagasaki; BMNH.

###### Remarks.

Morimoto et al. (2015) split *P.setosus* to two species, *P.setosus* and his newly described *P.squameus*. Morimoto studied only Japanese material, but he cited for both species the original distribution of *P.setosus* – Japan, Korea and China. Material cited in earlier literature must therefore be revised to check the identity of the specimens. The species can be distinguished by their elytral setae and also by the different shape of the spermatheca. However, we can not confirm that elytral setae are a stable distinguishing character, because material from places other than Japanese islands seems to be variable in this character, but the spermatheca seems to be useable. Based on the spermatheca, we can confirm the occurrence of the both species, *P.setosus* and *P.squameus*, in China.

##### Pseudocneorhinus squameus

Taxon classificationAnimaliaColeopteraCurculionidae

Morimoto, 2015

Figs 29 , 30 , 49

Pseudocneorhinus squameus Morimoto, 2015: 336 (original description); Alonso-Zarazaga et al. 2017: 403 (catalogue).
Pseudocneorhinus squamous (lapsus): Morimoto et al. 2015: 336 (Japanese fauna).

###### Material examined.

Other material. CHINA – **Beijing**; 1 ♀; Mentougou, Xiaolongmen [门头沟小龙门]; 39°57.6'N, 115°25.8'E; 1164–1210 m a.s.l.; 5 Jul. 2011; G.X. Qiao & J. Chen leg. [乔格侠, 陈军]; IZCAS, IOZ(E)1965222; 5 ♀♀; Xiaolongmen, Dongling Mountains, Liu Lang Yu; 39°58.2'N, 115°25.8'E; 1400 m a.s.l.; 15 Jun. 2001; Litter; J. Cooter leg.; BMNH; 1 ♀; Xiaolongmen, Dongling Shan; 39°57.688'N, 115°26.342'E; 1150 m a.s.l.; 11 Jun. 2004; J. Cooter leg.; swept by stream; BMNH; 1 ♀; Dongling Shan, 100 km W of Beijing; 1500 m a.s.l.; 12–15 Jun. 2000; Z. Jindra leg.; PKSC. – **Hebei Prov.** 2 ♀♀; Chengde, Wuling (shan) Mts., Longtan Scenic Spot; 40°35.72'N, 117°27.4'E; 1365 m a.s.l.; 8 Aug. 2016; P. Kment leg.; NMPC. – **Shanxi Prov.** 1 ♀; Lüliang Shan, road Fangshan – Jiaocheng, Hengjian env.; 1000 m a.s.l.; 9 Jun. 2000; Z. Jindra leg.; PKSC.

**Figures 25–30.**
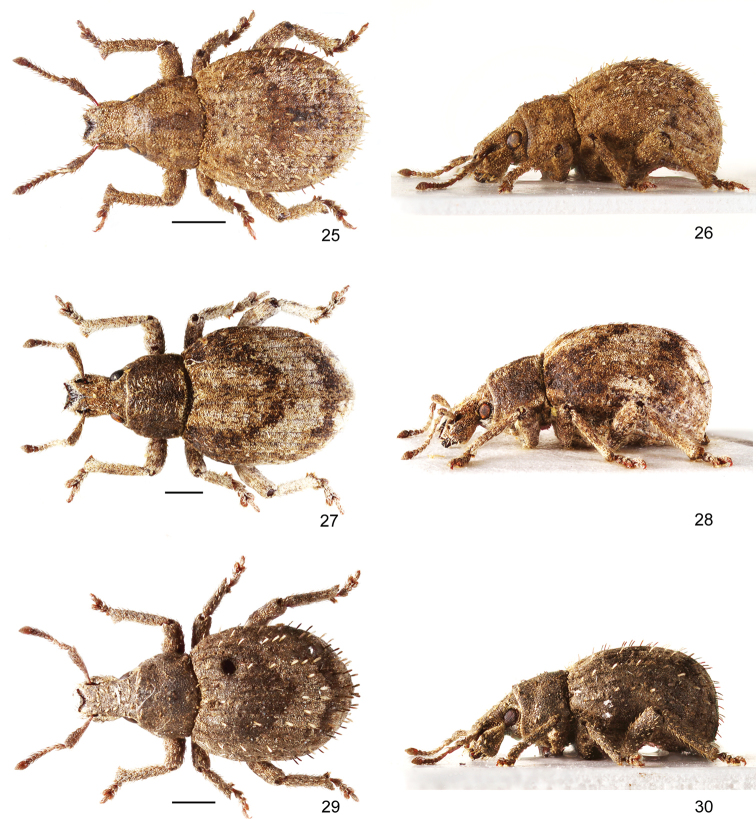
Habitus of *Pseudocneorhinus* species **25, 26***Pseudocneorhinusobesus*, female, dorsal and lateral view **27, 28***P.sellatus*, female, dorsal and lateral view **29, 30***P.squameus*, female, dorsal and lateral view. Scale bars: 1 mm.

##### Pseudocneorhinus squamosus

Taxon classificationAnimaliaColeopteraCurculionidae

Marshall, 1934

Fig. 50

Pseudocneorhinus squamosus Marshall, 1934: 6 (original description); Borovec 2009: 76 (check-list); Borovec 2013: 418 (catalogue); Alonso-Zarazaga et al. 2017: 403 (catalogue).

###### Type material examined.

Marshall (1934) based the description on specimens from “China: S. Kansu, 1 ♂, 1 ♀, 4.x. (Dr. Hummel)”. We studied one female, 3.47 mm long, from Marshall’s collection (BMNH), recently remounted and dissected by the second author. Lectotype ♀, here designated, with the labels: Cotype [printed, circular label with yellow margin] / Kina S. Kansu [printed] / Sven Hedins Exp. Ctr. Asien Dr Hummel [printed] / Pres. by Imp. Inst. Ent. B. M. 1934-130. [printed] / 4/10 [handwritten] / Pseudocneorrhinus squamosus Mshl. COTYPE ♀ [Marshall’s handwriting] / LECTOTYPUS Pseudocneorhinussquamosus Marshall, R. Borovec des. 2014 [red, printed].

##### Pseudocneorhinus subcallosus

Taxon classificationAnimaliaColeopteraCurculionidae

(Voss, 1956)

Figs 36 , 51

Cillirhopalus [sic] subcallosus Voss, 1956: 23 (original description).
Pseudocneorhinus subcallosus : Borovec 2009: 76 (check-list); Borovec 2013: 418 (catalogue); Alonso-Zarazaga et al. 2017: 403 (catalogue).

###### Type material examined.

Voss (1956) described this species based on six specimens from “Kwangtseh (10, 23–25.VII.1937), Shaowu (28.VII.1937)” without a type designation. We studied four of the specimens (ZFMK). Lectotype, here designated, with the labels: Kwangtseh-Fukien, J. Klapperich O, 23.7.1937 [violet, handwritten] / Callirhopalussubcallosus n. sp. [handwritten] / Holotypus Callirhopalussubcallosus n. sp. Voss 1949 [red, partly printed, partly handwritten] / LECTOTYPUS Pseudocneorhinussubcallosus Voss, R. Borovec des. 2019 [red, printed]. The other three have the following label data: one specimen with the same violet label as the holotype (without year) and labeled Paratypoid; one female with the same labels as the previous one but 24.7.1937; and one male with locality label Shaowu – Fukien, (500m) J. Klapperich 28.6.7.1937 and the same red “Paratypoid” label. Two specimens, one male and one female, were remounted by us. All these three specimens are designated here as Paralectotypes and provided with one more red and printed label PARALECTOTYPUS Pseudocneorhinussubcallosus Voss, R. Borovec des. 2019.

**Figures 31–36.**
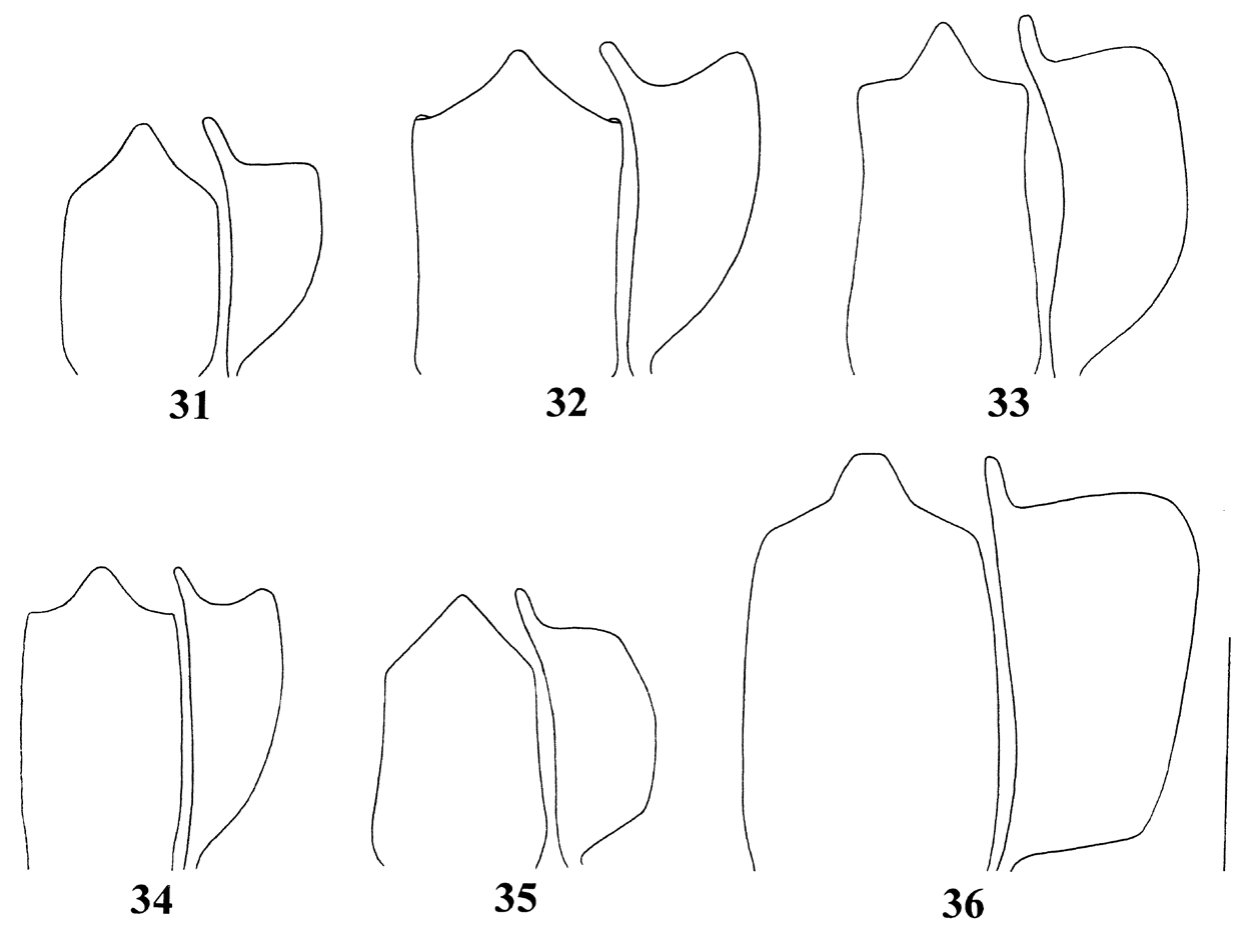
Penis in ventral and lateral view of *Pseudocneorhinus* species **31***Pseudocneorhinusangustus* sp. nov. **32***P.glaber* sp. nov. **33***P.setosicallus* sp. nov. **34***P.adamsi***35***P.bifasciatus***36***P.subcallosus*. Scale bar: 0.50 mm.

**Figures 37–51.**
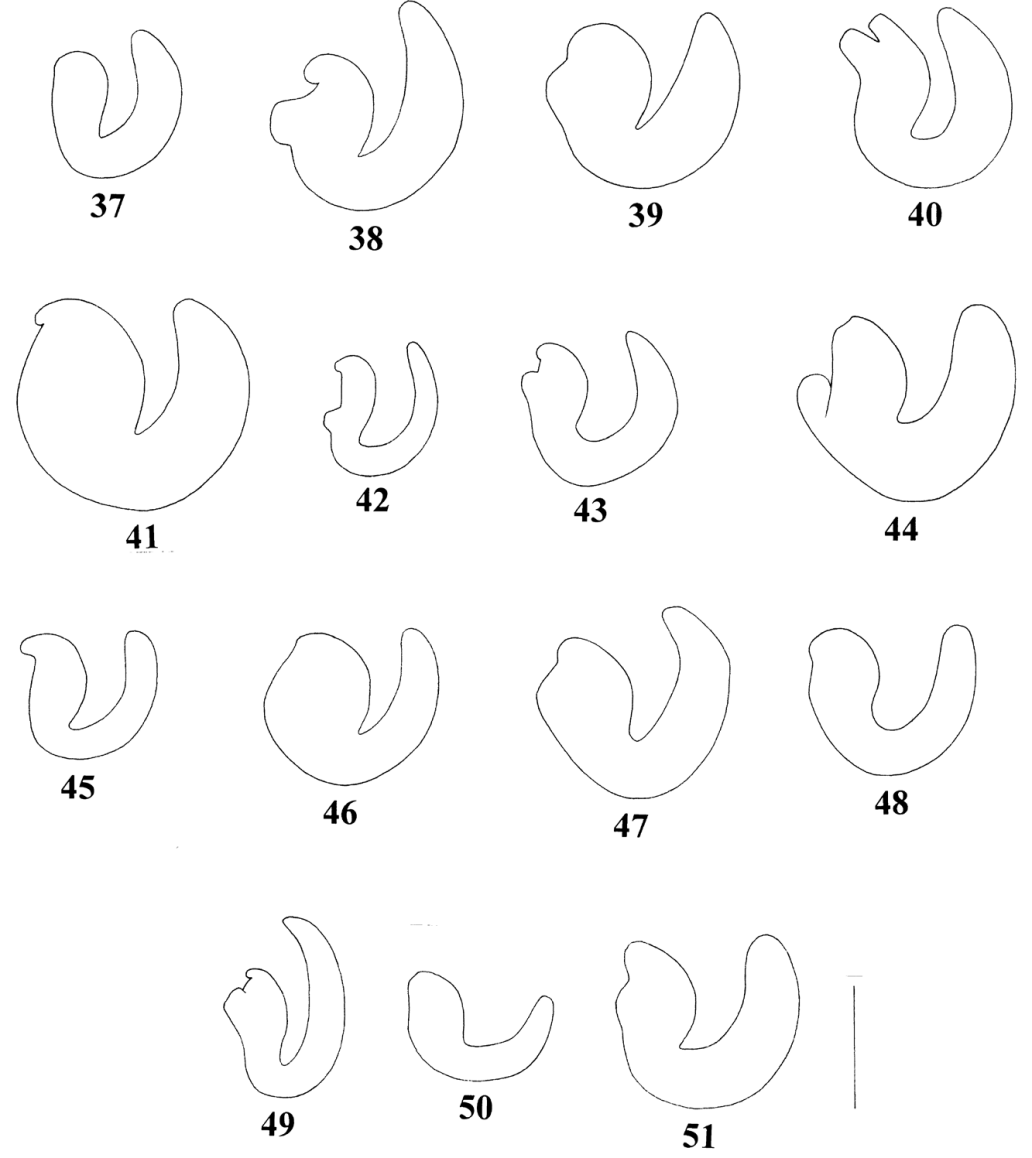
Spermatheca of *Pseudocneorhinus* species **37***Pseudocneorhinusglaber* sp. nov. **38***P.hlavaci* sp. nov. **39***P.setosicallus* sp. nov. **40***P.adamsi***41***P.alternans***42***P.bifasciatus***43***P.hirsutus***44***P.longisetosus***45***P.minimus***46***P.obesus***47***P.sellatus***48***P.setosus***49***P.squameus***50***P.squamosus***51***P.subcallosus*. Scale bar: 0.25 mm.

#### Key to the *Pseudocneorhinus* species

The following key separates the new species from all previously described ones. An asterisk (*) after the name means that species has not been studied by us and we know it only from the description.

**Table:** 

1	Metatibiae clearly denticulate on almost whole inner margin. Elytra with distinct longitudinal prominence distally at end of interval 2, visible mainly in lateral view	**2**
–	Metatibiae not denticulate on inner margin, only in *P.adamsi* and *P.longisetosus* with 3–5 minute denticles in apical half on inner face. Interval 2 of elytra without longitudinal prominence	**4**
2	Elytra in females as long as wide, in males slightly wider than long. Some intervals with two irregular rows of suberect setae. Size: 4.5–6.4 mm. Japan	*P.meshimanus* Morimoto*
–	Elytra in both sexes slightly longer than wide. Each interval with regular row of suberect setae	**3**
3	Space behind epistomal carina with round, iridescent scales. Elytra widest at anterior third. Size: 3.3–4.9 mm. China, Japan, Korea, Russia	***P.bifasciatus* Roelofs**
–	Space behind epistomal carina without round, iridescent scales. Elytra widest at middle. Size: 3.6–4.2 mm. Korea	*P.soheuksandoensis* Han & Yoon*
4	Dorsal part of body with inconspicuous, short, piliform, semiappressed greyish setae, hardly visible in lateral view, mainly at apical part of elytra (Figs 3–6). Size: 4.6–5.2 mm. China	***P.glaber* sp. nov.**
–	Dorsal part of body with conspicuous short to long, piliform to spatulate, semierect to erect setae, clearly visible also in dorsal view (Figs 7–30)	**5**
5	Scape distinctly widened distally, at apex distinctly wider than club and as wide as diameter of eye in lateral view	**6**
–	Scape moderately gradually widened distally, at apex as wide as or only slightly wider than club and conspicuously more slender than diameter of eye in lateral view	**7**
6	Raised elytral setae wide, subtriangular, truncate at apex. Rostrum at base abruptly enlarged. Funicle segment 3 1.1 × as long as wide; segments 4 and 5 isodiametric. Onychium equally long as tarsal segment 3. Size: 3.0–3.5 mm. China	***P.squamosus* Marshall**
–	Raised elytral setae slender, subspatulate, rounded at apex. Rostrum at base gradually enlarged. Funicle segments 3–5 wider than long. Onychium 1.2–1.3 × as long as tarsal segment 3. Size: 3.5–3.8 mm. China	***P.hirsutus* (Formánek)**
7	Raised elytral setae only on odd intervals or those on odd intervals more conspicuous, longer and distinctly denser (Figs 1, 11, 13). Odd intervals somewhat more elevated, at least on declivity	**8**
–	Raised elytral setae present equally on odd and even intervals (Figs 25–29). Odd intervals equally flat or convex	**12**
8	Elytra ovoid, widest in posterior third, shoulders not defined (Figs 1, 11, 13). Metatibial corbel with one long and one short mucro	**9**
–	Elytra oval, sides sub-parallel, widest at midlength, with distinct shoulders (Figs 9, 15). Metatibial corbel with two subequal mucros	**11**
9	Elytral setae inconspicuous, appressed, hardly visible mainly in lateral view. Rostrum 1.1 × as wide as long. Funicle segment 2 more robust, 1.4–1.6 × as long as wide. Size: 5.1–5.6 mm. China	***P.subcallosus* (Voss)**
–	Elytral setae conspicuous, perpendicularly erect, clearly visible in dorsal and lateral view. Rostrum 1.1 × as long as wide. Funicle segment 2 thinner, at least 1.8 × as long as wide	**10**
10	Smaller, 3.4–3.5 mm. Erect elytral setae half as wide as interval, spatulate (Fig. 1). Elytra more slender, 1.25–1.29 × as long as wide (Fig. 1). Onychium 1.1–1.2 × as long as segment 3. Scape with apex distinctly wider than club. Penis with larger, sharply pointed triangular apex (Fig. 31). China	***P.angustus* sp. nov.**
–	Larger, 4.2–5.8 mm. Erect elytral setae one fourth as wide as interval, lanceolate (Figs 11, 13). Elytra wider, 1.15–1.20 × as long as wide (Figs 11, 13). Onychium 0.8–0.9 × as long as segment 3. Scape with apex as wide as club. Penis with smaller, rounded triangular apex (Fig. 33). China	***P.setosicallus* sp. nov.**
11	Shoulders regularly rounded (Fig. 15). Elytral intervals 3 and 5 at declivity slightly enlarged and elevated. Funicle segments 3 and 4 isodiametric. Size: 4.2–5.8 mm. China	***P.alternans* Marshall**
–	Shoulders obliquely truncate (Fig. 9). Elytral intervals 3 and 5 at declivity distinctly enlarged with low longitudinal prominence. Funicle segments 3 and 4 1.2 × longer than wide. Size: 5.3–5.4 mm. China	***P.obliquehumeralis* sp. nov.**
12	Funicle segments 4 and 5 longer than wide	**13**
–	Funicle segments 4 and 5 wider than long	**15**
13	Rostrum almost parallel-sided (Fig. 25). Funicle segments 5–7 longer than wide. Size: 3.7–5.0 mm. China, Korea, Japan, Russia	***P.obesus* Marshall**
–	Rostrum with apex distinctly and regularly enlarged (Figs 11, 27). Funicle segments 5 and 6 isodiametric, segment 7 slightly wider than long	**14**
14	Raised elytral setae inconspicuous, semiappressed, shorter than half width of interval (Fig. 27). Rostrum isodiametric, with weakly rounded sides (Fig. 27). Epifrons with longitudinal slender carina in middle. Size: 5.2–6.7 mm. China	***P.sellatus* Marshall**
–	Raised elytral setae conspicuous, semierect, shorter than interval wide (Fig. 7). Rostrum 1.1 × as long as wide, with straight sides in basal half (Fig. 7). Epifrons without longitudinal carina. Size: 5.3–5.6 mm. China	***P.hlavaci* sp. nov.**
15	Elytra widest at middle (Fig. 23). Ocular lobes weakly developed. Size: 3.0–3.3 mm. China, Japan, Korea	***P.minimus* Roelofs**
–	Elytra widest behind middle (Fig. 29). Ocular lobes well developed	**16**
16	Setae on elytra squamiform, obtuse or truncate at tip, absent or much less numerous on intervals 4 and 6. Spermatheca with cornu long and slender, laterally extending beyond level of nodulus (Fig. 49). Size: 4.5–5.1 mm. China, Japan, Korea	***P.squameus* Morimoto**
–	Setae on elytra much narrower, acuminate, present on all intervals. Spermatheca with cornu more robust, not extending beyond level of nodulus (Figs 40, 44)	**17**
17	Metatibiae not denticulate on inner face. Epistome accompanied by narrow glabrous area. Size: 3.3–5.8 mm. China, Japan, Korea, Russia	***P.setosus* Roelofs**
–	Metatibiae with 3–5 minute denticles in apical half on inner face. Epistome accompanied by wide glabrous area	**18**
18	Epistome shorter, almost rectangular posteriorly, posterior corners shortly and narrowly rounded. Spermatheca with ramus slightly larger than nodulus, placed next to it (Fig. 40). Size: 4.1–5.7 mm. China, Japan, Korea	***P.adamsi* Roelofs**
–	Epistome longer, sharply triangular posteriorly. Spermatheca with ramus distinctly smaller than nodulus, placed at its base (Fig. 44). Size: 4.9–5.6 mm. Japan, Russian Far East	***P.longisetosus* Morimoto**

## Discussion

**Figure 52.**
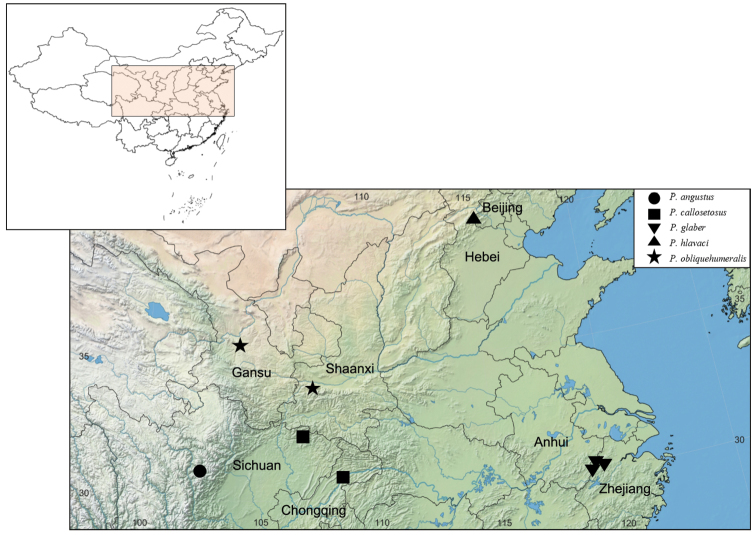
Geographical distribution of new species of *Pseudocneorhinus* in China.

There are 16 species of *Pseudocneorhinus* recorded from China, accounting for 84% of the species presently known in the Palaearctic Region. All species inhabit elevations between 240 and 3200 m; most of them were found around 1000 m. Ten species are Chinese endemics, except *P.alternans* and *P.sellatus* with, apparently, highly restricted distributions. Only five species are widely distributed between China and eastwards into the Korean Peninsula, the Russian Far East and Japan. We recognize two main distributional ranges in China. One is a longitudinally wide corridor from Heilongjiang to Fujian provinces in the Northeast and the eastern coastal areas. The other is in the Southwest, mainly southern Kansu, southern Shaanxi, Chongqing, and Sichuan provinces. All new species described herein have been discovered in mountainous localities (Fig. 52).

Marshall (1934) stated that *Pseudocneorhinushirsutus* was found at Kuku-Nor, Tibet. Alonso-Zarazaga et al. (2017) interpreted this locality as Xizang Autonomous Region. The specimens of *P.hirsutus* examined by us bear the label “China, THIBET, Kuku-Nor, 3200 m, 1898, Hauser lgt.” However, Kuku-Nor is the Mongolian name for Qinghai Lake, in Qinghai province. This means that Marshall (1934) referred to the Qinghai-Tibet Plateau rather than to a place in Xizang Autonomous Region. Consequently, the known occurrence of this species is corrected here to Qinghai, Kuku-Nor, which is possibly collected on the lakeside. Morimoto et al. (2015) reported *P.squameus* from China but gave no locality data from there. Here we confirm that *P.squameus* occurs in Beijing Municipality and Shanxi province. Other new records are Fujian province for *P.minimus* and Sichuan province for *P.sellatus*.

## Supplementary Material

XML Treatment for Pseudocneorhinus angustus

XML Treatment for Pseudocneorhinus glaber

XML Treatment for Pseudocneorhinus hlavaci

XML Treatment for Pseudocneorhinus obliquehumeralis

XML Treatment for Pseudocneorhinus setosicallus

XML Treatment for Pseudocneorhinus adamsi

XML Treatment for Pseudocneorhinus alternans

XML Treatment for Pseudocneorhinus bifasciatus

XML Treatment for Pseudocneorhinus hirsutus

XML Treatment for Pseudocneorhinus longisetosus

XML Treatment for Pseudocneorhinus minimus

XML Treatment for Pseudocneorhinus obesus

XML Treatment for Pseudocneorhinus sellatus

XML Treatment for Pseudocneorhinus setosus

XML Treatment for Pseudocneorhinus squameus

XML Treatment for Pseudocneorhinus squamosus

XML Treatment for Pseudocneorhinus subcallosus
